# Effective Exploration Behavior for Chemical-Sensing Robots

**DOI:** 10.3390/biomimetics4040069

**Published:** 2019-10-12

**Authors:** Kevin Nickels, Hoa Nguyen, Duncan Frasch, Timothy Davison

**Affiliations:** 1 Department of Engineering Science, Trinity University, One Trinity Place, San Antonio, TX 78212-7200, USA; dfrasch@trinity.edu (D.F.); tdavison@trinity.edu (T.D.); 2 Department of Mathematics, Trinity University, One Trinity Place, San Antonio, TX 78212-7200, USA; hnguyen5@trinity.edu

**Keywords:** chemotaxis, phototaxis, RapidCell, exploration, robot, *E. coli*, e-puck, navigation, obstacle

## Abstract

Mobile robots that can effectively detect chemical effluents could be useful in a variety of situations, such as disaster relief or drug sniffing. Such a robot might mimic biological systems that exhibit chemotaxis, which is movement towards or away from a chemical stimulant in the environment. Some existing robotic exploration algorithms that mimic chemotaxis suffer from the problems of getting stuck in local maxima and becoming “lost”, or unable to find the chemical if there is no initial detection. We introduce the use of the RapidCell algorithm for mobile robots exploring regions with potentially detectable chemical concentrations. The RapidCell algorithm mimics the biology behind the biased random walk of *Escherichia coli* (*E. coli*) bacteria more closely than traditional chemotaxis algorithms by simulating the chemical signaling pathways interior to the cell. For comparison, we implemented a classical chemotaxis controller and a controller based on RapidCell, then tested them in a variety of simulated and real environments (using phototaxis as a surrogate for chemotaxis). We also added simple obstacle avoidance behavior to explore how it affects the success of the algorithms. Both simulations and experiments showed that the RapidCell controller more fully explored the entire region of detectable chemical when compared with the classical controller. If there is no detectable chemical present, the RapidCell controller performs random walk in a much wider range, hence increasing the chance of encountering the chemical. We also simulated an environment with triple effluent to show that the RapidCell controller avoided being captured by the first encountered peak, which is a common issue for the classical controller. Our study demonstrates that mimicking the adapting sensory system of *E. coli* chemotaxis can help mobile robots to efficiently explore the environment while retaining their sensitivity to the chemical gradient.

## 1. Introduction

Autonomous systems that can leverage the sense of smell would be useful in many situations that are dirty, dangerous, or dull. Some examples include locating and rescuing victims trapped by rubble during a disaster, sniffing for illegal drugs or explosives, detecting and locating the source of chemical leaks on land or underwater, or even locating truffles in a forest. A robotic system that could quickly and reliably locate the region containing an odor would be a benefit in these, and other, situations.

A *taxis* is a movement toward, or away from, some stimulus such as light (phototaxis), airflow (anemotaxis), or a chemical (chemotaxis). Many organisms exhibit taxis behaviors, such as *Escherichia coli*, the silkworm moth *Bombyx mori*, and the dung beetle *Geotrupes stercorarius* [3]. Taxes have long been utilized as a control strategy in robotic systems. From Braitenberg’s simple vehicles [4] to an entire chapter of the canonical Springer Handbook of Robotics [5], taxes are one of a number of ways that biological systems have inspired roboticists. Some representative works are described below.

Biomimetic algorithms take inspiration from the physiology and biology of various organisms. Farrell et al. [6] take inspiration from the pheromone-tracing behavior of moths to control an underwater autonomous vehicle (UAV) tracing a plume to its source. Bau et al. [7] take inspiration from flying insects to track windborne odor plumes. Russell [8] moves underground, investigating taxes for the control of burrowing robots searching for buried chemical sources. Bremermann [9] cast chemotaxis as a potential solution to foraging optimization. Many researchers including Hossain [10,11,12] have applied different chemotactic control algorithms to robots. Other good reviews of projects comparing biomimetic control strategies can be found in [13] (2008) or more recently [14] (2012).

While pure chemotaxis works well for certain situations, roboticists also experiment with a variety of hybrid schemes, sometimes combining biomimetic algorithms with those derived from artificial intelligence research. For example, Nurzaman et al. [15] start with biological chemotaxis and Levy walks individually, then study the combination of these behaviors in simulation and on a mobile robot using sound waves as a chemical proxy. Grasso et al. [16] combine mean flow and chemical detection strategies to enable RoboLobster to orient itself to a chemical source in turbulent flow.

Another group of researchers eschew direct biomimicry and attack the problem with analytic algorithms drawn from autonomous vehicles and artificial intelligence research [6]. Farrell et al. [6,17,18] note that in water and other high-Reynolds-number fluids, chemical detection is commonly binary—a chemical is detected or not detected at any point in time. They develop algorithms for a UAV to home in on a chemical plume emitted into ocean water. Zhang et al. [19] give a survey of strategies for chemical source localization in air, and derive a multi-stage algorithm for tracing plumes upwind. Ji-Gong et al. [20] use a three-stage process including a history of air flow directions over time to implement a path planning method to search an odor source, using range data detected by the robot to dynamically modify the planned path.

In recent years, many researchers have again drawn from nature and tackled the problem from the viewpoint of *swarm robotics*, where multiple robots are cooperatively searching for an attractant. Yang et al. [21] subdivide the search space using a Voronoi tessellation and search each cell in the tessellation using bacterial-style chemotaxis. Marjovi et al. [22] study optimal swarm formations for plume-finding, and present a set of virtual forces that allow several robots to line up in an equi-spaced diagonal line while also avoiding obstacles. Marques et al. [23] use a particle swarm to simulate a mass of small robots tracing an upwind odor source. Swarms of robots can also be controlled with particle swarm optimization (PSO) [23], gray wolf optimizer [24], or combinations of these [25,26]. Zarzhitsky et al. [27] leverage computational fluid dynamics to develop an algorithm for a particle swarm simulation that outperforms biomimetic algorithms. Turduev et al. [28] compare decentralized and asynchronous particle swarm optimization, bacterial foraging optimization, and ant colony optimization to build a 2D map of the concentration of ethanol gas in an environment. Yang et al. [12] draw from foraging algorithms to study target trapping.

Some projects directly compare methods drawn from various organisms in a particular application. In a classic work, Russell et al. [3] compare chemotaxis algorithms inspired by the bacterium *E. coli*, the silkworm moth *Bombyx mori*, and the dung beetle *Geotrupes stercorarius*, as well as a gradient-based algorithm developed by the authors. They conclude that each is best in different situations.

We define a basic chemotaxis algorithm as consisting of a gradient ascent with some probabilistic elements that vary the mix between running and tumbling motion. “Running” is when the agent moves in a straight line (as when ascending/descending the gradient), and “tumbling” is when it turns randomly to point in a different direction. We recognize that basic chemotaxis algorithms are good for finding the local maxima of chemical concentrations, under the assumption that the agent is able to make an initial detection of the chemical. However, if the goal is to do a more complete exploration of the full area with detectable chemical concentration, basic chemotaxis tends to be ineffective. The agent will often get stuck at a local maximum, or become “lost” in areas of zero or low chemical concentration and become unable to find the chemical even after extended periods of time. Given these issues with basic chemotaxis algorithms, we recognize the need for a more sophisticated algorithm for mobile robots whose goal is to both find the source of a chemical (the point or points of highest concentration) and also explore the full area where the chemical is detectable.

In previous work we used an algorithm called RapidCell [1], originally developed to describe the interactions of chemotaxis proteins inside *E. coli* cells. We chose to incorporate RapidCell because it provides a more realistic representation of the run/tumble behavior of *E. coli*, and thus can be used to create robotic behaviors that are more sophisticated than those of basic chemotaxis. In [29] we coupled RapidCell with the method of regularized Stokeslets [30] to simulate *E. coli* chemotaxis in a viscous fluid. In subsequent work [31], we couple RapidCell with the lattice-Boltzmann method [32] to simulate how engineered micro-particles utilize *E. coli*’s biased random walk to detect the location of high chemical concentration contained in a confined zone with a narrow inlet or concentric multiringed inline obstacles, mimicking tumor vasculature geometry. In [33], instead of imposing static concentrations as in [29,31], we implement dynamic multiple concentrations of different chemicals to show how the chemotactic behavior changes over time as the cells disturb and mix the chemicals.

The results of these previous efforts lead us to hypothesize that a chemical-sensing mobile robot operating with a motion algorithm utilizing RapidCell would have the following advantages over basic chemotaxis:

- The robot will more completely explore the environment in regions both with and without detectable chemical.
- The robot will be less likely to get “lost” in areas of low or zero concentration.
- The robot will be less likely to get stuck at local concentration maxima.

In this work, we augment the RapidCell approach with a variation aimed at avoiding collisions with obstacles in the environment, implement the algorithm in simulation and on a tabletop robot (using phototaxis as a surrogate for chemotaxis), and compare with a basic chemotaxis algorithm described in [2] using several metrics. In Section 2, we describe the experimental scenarios, the control algorithms in use, and our metrics for success for the robot. Then, in Section 3 we provide representative and summary results of the simulations and experiments conducted. We also provide a detailed discussion of the results in Section 3. Finally, in Section 4 we draw some conclusions about the pros and cons of the algorithms under consideration.

## 2. Materials and Methods

Our goal in this work was to compare the performance of a basic chemotaxis algorithm with a novel motion algorithm that incorporates RapidCell. We applied these algorithms in a mobile robot exploring an environment with a chemical concentration that varied with position. We tested both algorithms in simulation and on a real robotic system, in environments with and without obstacles. In addition, we explored three different types of chemical concentration distribution:

1. A homogeneous environment with no chemical effluent (we define *effluent* as a discharge of a specific—desired or undesired—chemical into an area).
2. An environment with a single chemical effluent.
3. An environment with three chemical effluents.

**Figure 1:**
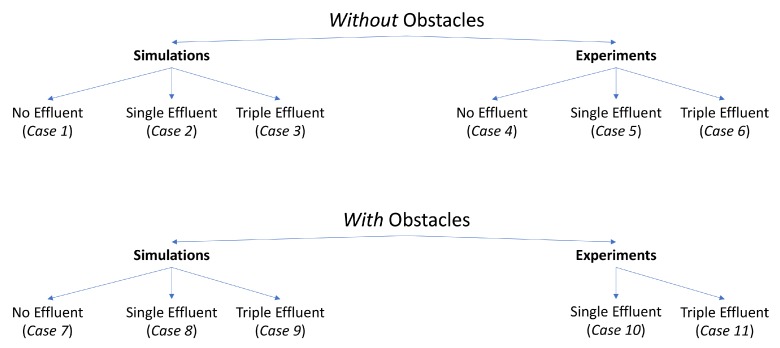
Different scenarios in this work.

The simulations and experiments are detailed in Figure 1. Notably absent from this set is an experiment without effluent, but with obstacles. The minimal differences in qualitative behavior between the experiment in the single effluent or triple effluent environment without obstacles (Cases 5 and 6) and with obstacles (Cases 10 and 11), and the very limited exploration about the initial positions in the relevant simulations (Cases 1 and 7), led us to choose to not conduct this set of experiments.

The goal of our work was not (yet) to create or study a robotic system that actually samples a chemical plume using some type of sensor. So, as an analog to such a system we used a small tabletop robot equipped with an optical sensor that detects the brightness of the surface below it. To imitate the chemical gradients, grayscale gradients were printed on posters on top of which the robot operated, as seen in many other works (e.g., [34]). The robot itself was assumed to be 2 μm across, with the sensor cluster located 1 μm from its centroid at the front end. Constant factors were used to scale between the simulations and experiments. A square arena of 200 μm×200 μm with $A_{tot}=$ 40,000 $\mu m^{2}$ was used for all simulations and experiments.

**Figure 2:**
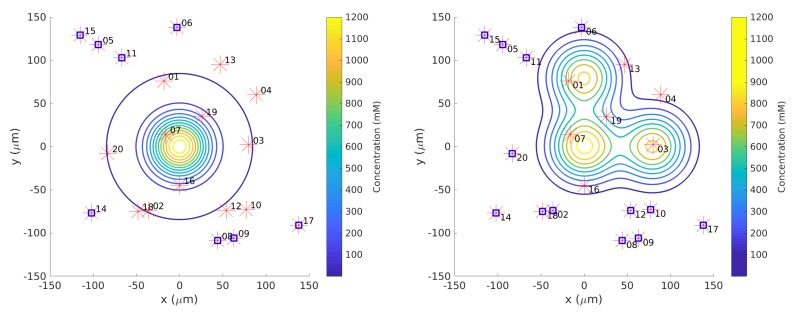
Twenty initial positions for all simulation trials marked as red asterisks. Some trials began in detectable concentration (C>1mM—the outer blue circle) and some began outside. Positions marked with blue squares on top of the asterisks fell outside the region covered by the grayscale poster. Therefore, the remaining positions were used for all experimental trials.

Twenty initial positions were chosen from a uniform distribution within the arena. The same initial positions were used for each simulation. In the single-effluent experiments, only 12 of the 20 initial positions were in the region covered by the grayscale poster, so those 12 were used for Cases 5 and 10. Figure 2 shows the initial positions as well as the region of detectable concentration (concentrations above 1 mM) for the single-effluent environment. In the three-effluent experiments, only 7 of the 20 initial positions were in the region covered by the grayscale poster, so those 7 were used for Cases 6 and 11. Figure 2 also shows the initial positions as well as the region of detectable concentrations (concentrations above 1 mM) for the triple-effluent experiment. These were the largest posters (approx 91 cm${}^{2}$) that would fit in the experimental environment. In each case the robot had no prior knowledge of the chemical present or the type, location, or presence of obstacles.

### 2.1. Chemical Effluent Models

In this section we give details about the chemical concentration profiles that were modeled.

**Homogeneous/No Effluent Environment**: As a control, each algorithm was first tested in a homogeneous environment with no detectable chemical present. In the simulation (Cases 1 and 7), the function C(x,y) which represents a chemical concentration was set to 0 at every (x,y) location. In the experiment (Case 4), a white laminated poster with the size of the arena was created to represent this environment. Due to noise and other non-ideal experimental conditions (room lighting, etc.), C was low but not equal to zero at different locations on the poster.

**Figure 3:**
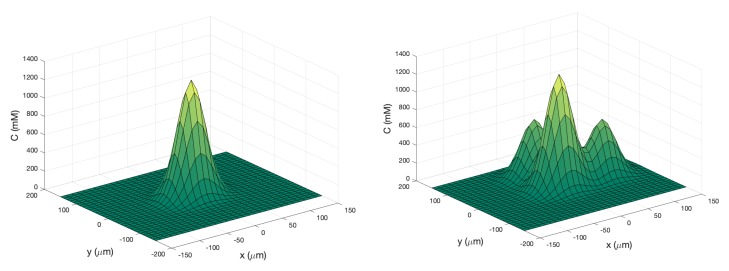
**Left**: Surface plot of static function (Equation (1)) to describe the single-effluent chemical concentration in the arena. The peak of this one effluent is at the center (0,0)μm. **Right**: Surface and contour plots of static composite function to describe the three-effluent chemical concentration imposed on the arena. The highest peak is located at the center (0,0)μm while the two local peaks are at (0,80) μm and (80,0) μm.

**Single Effluent**: In this environment, there was a single chemical effluent centered in the arena. In the simulation (Cases 2 and 8), the concentration detected by the robot was described with the static function shown in Equation (1) with experimentally determined parameters M = 4000 (mM), D = 0.0125 ($\mu m^{2}/s$), and $t_{s}$ = 2000 (s), centered at (0,0). This function is an approximation for a point source of chemical effluent [35]. Other choices, for example, a Gaussian plume model [36], could also be used. Figure 3 (left) shows a surface plot of the fixed time function with these parameters. The concentration C was truncated at 1 mM, the assumed minimum value detectable by a chemical sensor.

(1) $$C\left(x,y\right)=\frac{M}{4\pi Dt_{s}}e^{\left(\frac{-x^{2}-y^{2}}{4Dt_{s}}\right)}\;[\mathrm{mM}]$$

**Figure 4:**
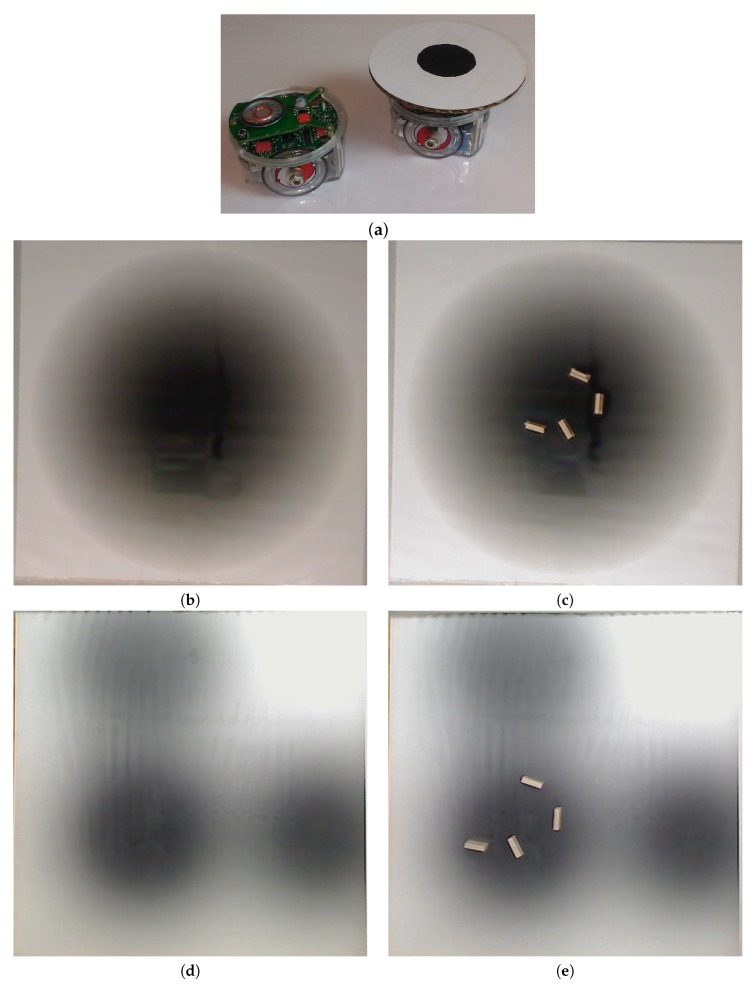
The e-puck robot used in the experiment can be seen in (**a**), with and without the “lid” used to make localization easier. The robots were tested on a grayscale gradient poster. Tests were done (**b**,**d**) without additional obstacles and (**c**,**e**) with obstacles in the middle of the poster. (**b**,**c**) represent the single-effluent environment while (**d**,**e**) represent the triple-effluent environment.

In the experiment (Cases 5 and 10), a grayscale circular pattern darker in the center was printed on a laminated poster to represent the environment (see Figure 4b). A reflective sensor in the robot was used to measure the reflected light at a given position. Then, the raw data from the sensor was mapped to Equation (1) as described in Section 2.5.

**Triple Effluent**: In the third environment, the chemical concentration was a composite of three functions: two similar functions to Equation (1) with the smaller parameter value M = 2000 mM and centers at (80,0) and (0,80) were added to the original function. Figure 3 (right) shows a plot of this concentration, which models three effluents in the simulations of Cases 3 and 9.

### 2.2. Obstacles

**Figure 5:**
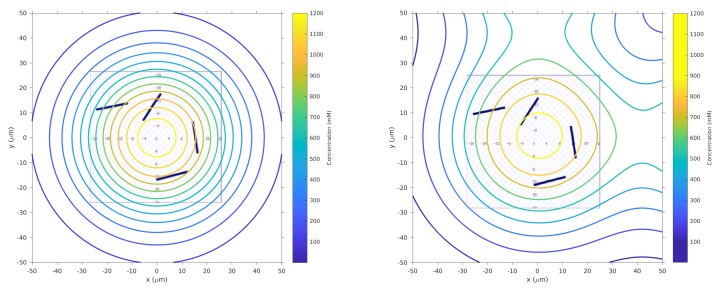
Obstacles shown as black rectangles are overlaid with the contour plots of the concentration in Figure 3. Note that this is a zoomed-in view of the domain to clearly show the obstacle locations.

We added small 10 μm×2 μm obstacles at the locations shown in Figure 5 for all simulations and experiments, with some minor variations in the orientation of the individual obstacles.

### 2.3. Control Algorithms

This section describes the basic chemotaxis algorithm and the RapidCell *E. coli* model/algorithm used in this work.

**Figure 6:**
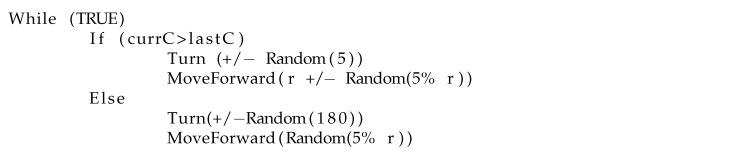
Basic chemotaxis algorithm, adapted from [2]. Angles are in degrees, and *r* represents the length of a typical run.

**Basic Chemotaxis Algorithm**: For comparison to the RapidCell model described below, we implemented the simple chemotaxis algorithm described in [2], in turn adapted from [37]. The algorithm, described in Figure 6, randomly turns with a bit of forward motion when the concentration is decreasing, and moves forward with a bit of random turning when the concentration is increasing. It is, in general, a straightforward hill-climbing algorithm, and tends to hover in a very small region around the first local maximum detected.

**RapidCell Algorithm**: In the presence of a spatial gradient, *E. coli* move towards an attractant and away from a repellent. Changes in the detected chemical concentration over time affect the interactions of chemotaxis proteins inside the cell, which control the flagellar motors to rotate counter-clockwise (CCW) or clockwise (CW). If all the flagella rotate CCW, they will form a bundle and push the cell to *run* forward. If the cell wants to *tumble*, the flagellar bundle can be disintegrated by having at least one flagellum switch from CCW to CW. The tumbling motion allows the cell to re-orient itself in a different direction. In the absence of a chemical gradient, the cell performs a random walk consisting of short episodes of smooth runs terminated by tumbles. However, when molecules of attractants or repellents start binding to the receptors on the cell membrane, the cell will evaluate changes in the amount of concentration. Using its cell signaling pathway, the cell can integrate the binding information it receives into a sequence of chemotaxis-protein interactions that will control the rotations of the flagellar motors and hence give the probability for the cell to run or tumble. So, the cell signaling pathway can be thought of as a temporal mechanism which allows the cell to compare their current binding state with the previous ones. An increase in the fraction of an attractant binding to receptors will raise the probability of CCW rotation, which will lead to extended runs (biased random walk). Note that the cell signaling pathway and resulting behavior are different when the cell encounters a repellent instead of an attractant. In this work, we do not consider repellents.

**Figure 7:**
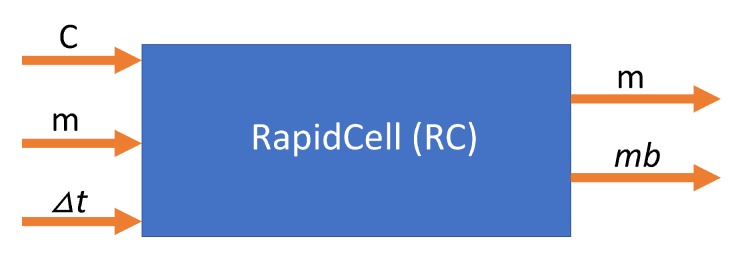
RapidCell model as a function of current concentration C, methylation m, and time interval Δt. The results are the updated methylation m and motor bias mb.

The RapidCell model [1] was originally developed to describe the interactions of these chemotaxis proteins inside *E. coli* cells when sensing attractants. If we consider the RapidCell model as a function (see Figure 7), the inputs are the concentration at the sensor of a robot (C), the methylation level (m) in the previous time step, and the difference in time (Δt) from the last computation. Methylation can be considered a memory mechanism in *E. coli*, as it builds up the more time the cell spends in an area of detectable concentration. The outputs are the updated methylation level, and the motor bias mb. The motor bias mb represents a probability of running; its values range from 0 to 1, with higher values corresponding to a greater likelihood of running motion of bacteria. Further details regarding the description of the RapidCell model and model parameters can be found in [1] and Appendix A.

**Obstacle Detection and Avoidance**: Obstacle detection and avoidance behavior was added to both the basic and RapidCell chemotaxis algorithms. At each run–tumble decision, a forward-facing obstacle sensor is checked. If an obstacle is in front of the robot, a random turn is performed regardless of the current concentration. If no obstacle is found, the existing chemotaxis is performed. This hierarchical decision-making is reminiscent of many multi-layer controllers, including the classical subsumption architecture [38], and was not expected to qualitatively alter the behavior of either controller.

### 2.4. Simulation Setup

The basic chemotaxis and RapidCell-based controllers were first implemented in the Player/Stage robotic simulation environment [39]. A spatial scale factor of 1 μm = 1 m was used in simulation due to the resolution limits built into Player/Stage. Each controller was allowed to run for 10,000 control iterations in each trial.

### 2.5. Experimental Setup

In the experimental cases for the single-effluent and triple-effluent environments, a grayscale pattern was printed on a laminated poster shown in Figure 4b,d. For the environment with obstacles, Figure 4c,e show how the obstacles were added to the arena. In addition, walls were placed as a barrier around the arena to prevent the e-pucks from running off the testing table since they are recognized as obstacles for the robots.

In order to test the performance of each controller on a real robot, we then paired the basic and RapidCell controllers with a custom e-puck driver. E-pucks are small, fist-sized two-wheeled robots shown in Figure 4a and introduced in [40]. They are equipped with a number of sensors, including three TCNT1000 [41] infrared-pair sensors on the bottom near their front. These sensors are used to detect the reflectivity of the surface below the e-puck. White and highly reflective surfaces read as high values for these sensors, while darker surfaces read low.

The center light sensor was used to perform chemotaxis in these experiments. The sensor reading was calibrated to mimic the concentration detection sensors of *E. coli* via Equation (2) in Cases 5 and 10, and Equation (3) in Cases 6 and 11. The differing ink darkness of the posters led to a different mapping between “darkness” and “concentration”:

(2) $$C\left(s\right)=2\times e^{0.008\times (1000-s)},\;[\mathrm{mM}]$$

**Figure 8:**
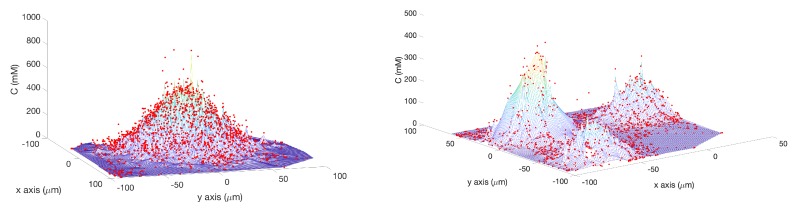
**Left**: Interpolated surface of concentration C to show how the raw sensor reading (shown as red dots) was calibrated via Equation (2) at different locations on the single-effluent poster shown in Figure 4b. **Right**: Interpolated surface of concentration C to show how the raw sensor reading (shown as red dots) was calibrated via Equation (3) at different locations on the triple-effluent poster shown in Figure 4d.

(3) C(s)=1−0.001×s,            [mM]

where *s* is the raw sensor reading (0–1024). Therefore, darker spots towards the center of the effluent had low values for the raw sensor reading (*s*) which represent high chemical concentration (C). The spatial variation of C over several experimental runs in the single-effluent and triple-effluent environments can be seen in Figure 8.

The e-puck is also equipped with eight outward-looking infrared sensors for obstacle detection and avoidance. The front four sensors were used for this work. The effluent image was approximately 200 μm in diameter, and the poster approximately 90 cm square, so a scaling factor of 1:4500 was assumed between simulation and experimentation for both experiments.

The basic chemotaxis algorithm was allowed to run for 100 iterations, and the RapidCell algorithm for 500 iterations, to give each time to explore any effluent present. It was observed that in every case when the basic chemotaxis algorithm found detectable chemical, it converged on a local maximum by 100 iterations. An overhead webcam was used to take images of the arena after each control cycle. Each cycle consisted of 0.5 s of run-time plus 5 s in order to give the overhead webcam time to take an image and save it to hard disk. With the lid shown in Figure 4a, a simple two-dimensional template matching was used to locate the e-puck in each image, and an orthographic projection was used to localize the robot within the arena, similar to [42].

For each cycle of the basic chemotaxis control loop, the robot took a sample of the chemical concentration. The e-puck then used Equation (2) or Equation (3) to map the grayscale sensor input to a particular chemical concentration value, while the simulated robot determined the concentration through an a-priori fixed function and the current position obtained from the simulator. The concentration was then sent to the chemotaxis model, which generated a Turn and MoveForward velocity in the basic chemotaxis algorithm presented in Figure 6.

For the RapidCell control loop: when the controller was started, the robot took a large number of samples of the initial concentration of the chemical and passed it through the RapidCell model. This allowed the methylation m, and other parameters to adjust to the initial environment. After this initialization, the robot began the RapidCell control loop. In each cycle of the loop, the robot took a sample of the chemical. The concentration was then sent to the chemotaxis model, which generated an updated methylation value m and motor bias mb, which was a number between 0.0 and 1.0. The motor bias was treated as the probability of a run on that control cycle.

### 2.6. Measurements of Success

We assumed the goal of the robot was to explore all locations within the region of interest with detectable chemical concentration. We propose the following measurements of success as we compared the results from the basic chemotaxis algorithm and the RapidCell algorithm.

There are several regions that we reference below, which we formally define here:

**Definition** **1.** *The* Region of Interest *is the entire rectangular area from* −100 μm *to* 100 μm *in both horizontal and vertical directions, possibly containing chemical effluent. The total area of this region is $A_{tot}$ =* 40,000 *$\mu m^{2}$.*

**Definition** **2.** *The* Capture Region *is, for the RapidCell controller, an area the robot will remain in once it has entered. Informally, the robot is* captured *in this region. It can be thought of (roughly) as the region where the effluent concentration is detectable.*

**Definition** **3.** *The* Peak Region *is the area immediately adjacent to (within 1–2 body lengths of the robot) the maximum concentration of effluent.*

We also define a *replicate* as an iteration of the simulation or experiment with a particular initial position (all the initial positions are shown in Figure 2).

**Figure 9:**
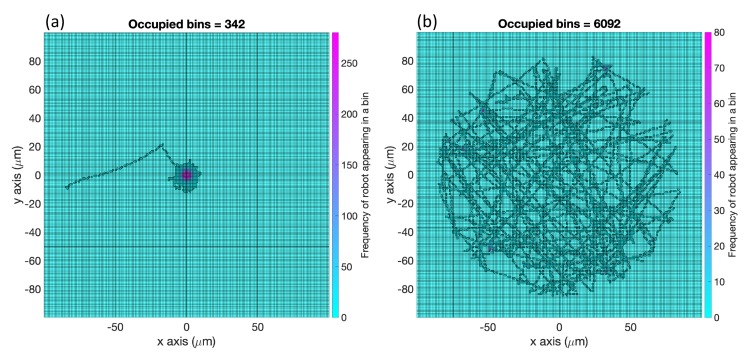
Explored bins are those with detected concentration that were visited. This simulation environment had one effluent concentration in the center (0,0) of the region of interest (as shown in Figure 3) and contained no obstacles. The basic chemotaxis algorithm (**a**) did not explore as much area (average percentage (AP) = 0.86%) as the RapidCell model (**b**, AP = 15.23%).

**Average Percentage (AP)**: For each replicate of a case described in Figure 1, we divided the region of interest into square bins of dimensions 1 μm×1 μm. Then, we recorded the number of bins with detectable concentration that the robot had visited for the cases with effluent. In the environment with no effluent, we simply counted all the bins the robot had visited. The bin was recorded only at the discrete timestep and not along the path the robot took between timesteps. We defined those bins as *explored*; see Figure 9 for an example. Then, we computed the percentage of the explored bins from the total number of bins $A_{tot}$ and took the average of these percentages (AP) from all the replicates of this case. The performance of all the cases in Figure 1 using the two controllers with respect to this AP measure are summarized in Table 1.

**Success Rate (SR)**: Due to the randomness in both algorithms and the existence of some initial positions outside the region of detectable chemical in the single effluent environment, we propose the success rate, which is the percent of replicates with positive concentration at the terminus of their run over the total number of replicates for each case and each controller. The higher the success rate, the better the controller in finding locations of positive concentrations in the region of interest without getting completely lost. Performance with respect to this measurement is summarized in Table 2.

## 3. Results and Discussion 

Both the basic chemotaxis and RapidCell controllers were exercised in simulation and on the table-top robots. This section describes the results of those simulations and experiments.

### 3.1. Simulation without Obstacles

In the first set of simulations (Cases 1–3 in Figure 1), the robot was placed according to the 20 initial positions described in Figure 2. No obstacles were placed in the environment with the robot.

**Figure 10:**
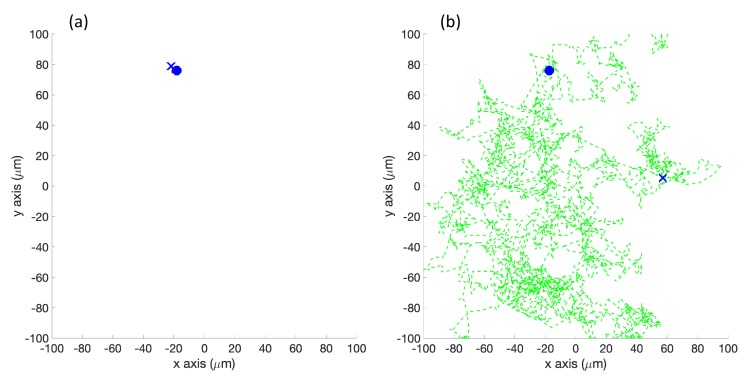
Case 1: Without obstacles, in the homogeneous/no effluent environment, typical trajectories of the simulated robot running (**a**) the basic chemotaxis controller, and (**b**) the RapidCell chemotaxis controller. In each trajectory, the initial position is shown as a blue circle and the terminal position is shown as a blue “X”. Positions corresponding to concentrations less than 1 mM are shown in dashed green, otherwise shown in red. The detected concentration was zero in both controllers for this environment.

**Simulation in Homogeneous/No Effluent Environment (Case 1)**: In the homogeneous environment, the simulated robot with the basic controller continually tumbled, with only a 5% forward motion on each control cycle. The resultant tracks, an example of which is shown in Figure 10a, show the limited degree of exploration that occurred with the basic chemotaxis algorithm in the absence of detectable concentration. In Figure 10b, the RapidCell chemotaxis algorithm performed an unbiased random walk, exploring a larger portion of the environment than the basic algorithm.

**Figure 11:**
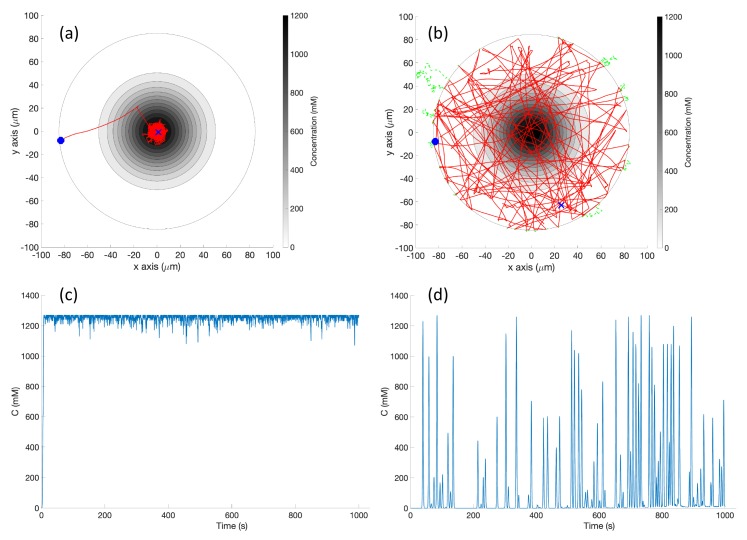
Case 2: Without obstacles, in the single-effluent environment, typical trajectories of the simulated robot running (**a**) the basic chemotaxis controller, and (**b**) the RapidCell chemotaxis controller. The contour plot represents the concentration distribution shown in Figure 3 (left) where the outmost level curve represents the concentration at 1 mM. In each trajectory, the initial position is shown as a blue circle and the terminal position is shown as a blue “X”. Positions corresponding to concentrations less than 1 mM are shown in dashed green, otherwise shown in red. The recorded concentrations associated with each scheme are shown in (**c**,**d**) respectively.

**Simulation in Single-Effluent Environment (Case 2)**: In the single-effluent case, when the robot encountered a detectable chemical, it quite efficiently moved toward the peak of the effluent using the basic chemotaxis algorithm, as shown in the trajectory in Figure 11a. It then proceeded to tumble in the small area (relative to the RapidCell controller) about the peak region of the chemical.

Figure 11b shows an example trajectory of the robot using the RapidCell chemotaxis algorithm in the same environment. From its initial position the robot performed biased random walk towards the center of concentration. The robot continued in approximately a favorable direction as long as it detected chemical, then rebounded randomly within the capture region defined in Section 2.6. When it moved outside the limit of detectable chemical (shown by the green trajectories), it tumbled until it detectd the chemical again.

Figure 11c demonstrates that the basic controller was very effective at remaining near the peak concentration once it found it. Comparatively, the detected concentration fluctuated much more in Figure 11d because the RapidCell algorithm did not become stuck at the peak concentration and more thoroughly explored the environment. The capture region from the RapidCell controller was significantly larger than the peak region identified by the basic controller.

**Figure 12:**
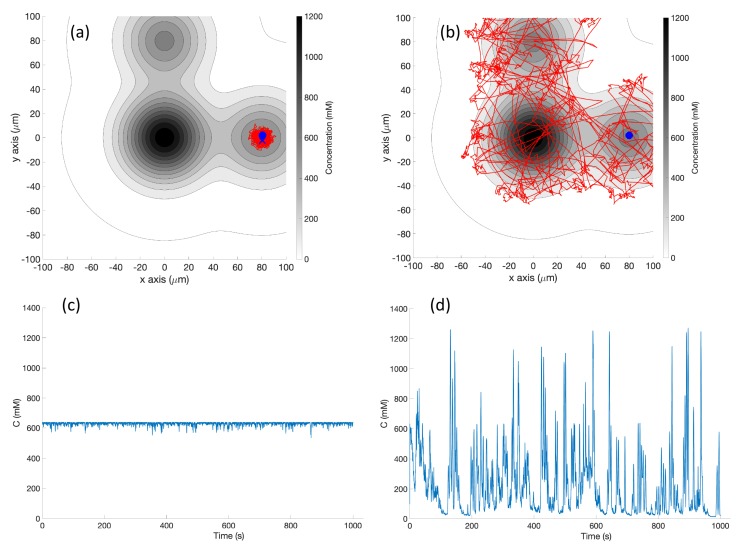
Case 3: Without obstacles in the environment and with triple effluents, the trajectories of the simulated robot running (**a**) the basic chemotaxis controller on the xy plane, (**b**) the RapidCell chemotaxis controller. (**c**,**d**) show the recorded concentrations associated with each scheme, respectively. The contour plot represents the concentration distribution shown in Figure 3 (right) where the outmost level curve represents the concentration at 1 mM. The same region of interest of 200 μm×200 μm was used for this environment. Thus, only trajectories within that region of interest are plotted.

**Simulation in Triple-Effluent Environment (Case 3)**: In the multiple effluent case in Figure 12a, if the initial position was outside the detectable limit of the basic chemotaxis controller, the robot often never found the chemical. The concentration profile for this case can be found in Figure 3 (right). However, if the robot found chemical, it climbed the gradient toward the closest local maximum, as expected for the basic controller.

Figure 12b shows the trajectory of a single representative replicate of the simulation using the RapidCell chemotaxis algorithm. Note that the behavior was quite random, but the same patterns of wide exploration could be observed. After approaching the capture region using biased random walks, the robot performed random walks inside the region, exploring the area with detectable concentration until the simulation ended.

The detected concentrations for the basic and RapidCell algorithms are plotted in Figure 12c,d, respectively. The robot operating with the basic algorithm got stuck at a local maximum at location (80, 0) μm (in this case, not the global maximum at location (0, 0) μm). In contrast, the RapidCell algorithm was able to explore the areas of all three effluents.

### 3.2. Experiments without Obstacles

The same controllers (basic and RapidCell) used with the Player/Stage simulation were paired with an e-puck mobile robot for experimental evaluation. Proximity sensors were used to avoid walls bounding the arena, in order to ensure the robot did not fall off the table top.

**Figure 13:**
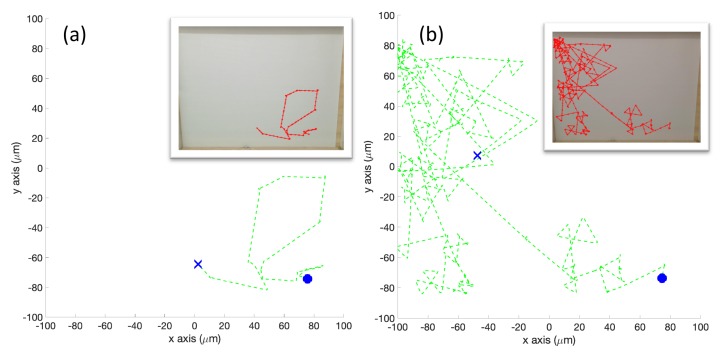
Case 4: Without obstacles, in the homogeneous/no effluent environment, typical trajectories of the e-puck robot running (**a**) the basic chemotaxis controller, and (**b**) the RapidCell chemotaxis controller. In each trajectory, the initial position is shown as a blue circle and the terminal position is shown as a blue “X”. Positions corresponding to concentrations less than 1 mM are shown in dashed green, otherwise shown in red.

**Experiments with Homogeneous/No Effluent Environment (Case 4)**: A plain white laminated poster with no black ink was used for the homogeneous experiments. Figure 13 shows a single representative run in this environment. The basic chemotaxis controller explored more compared to the simulation (Case 1), but the experimental RapidCell controller clearly explored more than the basic controller.

**Figure 14:**
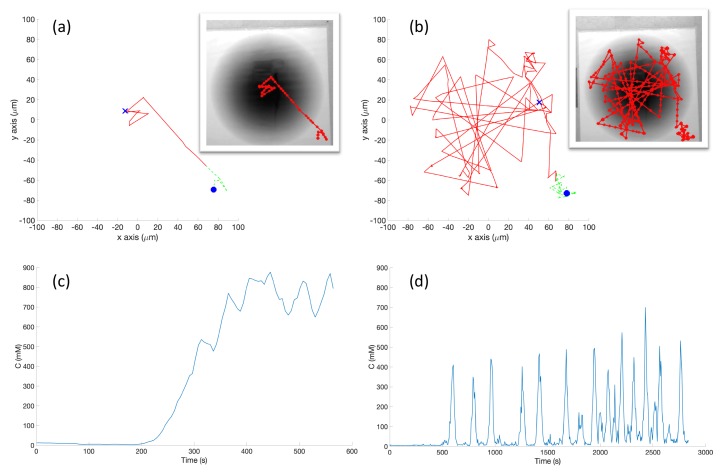
Case 5: Without obstacles, in the single-effluent environment, typical trajectories of the e-puck robot running (**a**) the basic chemotaxis controller, and (**b**) the RapidCell chemotaxis controller. In each trajectory, the initial position is shown as a blue circle and the terminal position is shown as a blue “X”. Positions corresponding to concentrations less than 1 mM are shown in dashed green, otherwise shown in red. The recorded concentrations associated with each scheme are shown in (**c**,**d**) respectively.

**Experiments with Single-Effluent Environment (Case 5)**: The experimental results were similar to the corresponding simulation results (Case 2). The basic controller generally traveled toward darker regions and explored the region in close proximity to the single peak. The RapidCell controller on the other hand explored the entire region of detectable intensity after encountering detectable chemical. Figure 14 plots one representative replicate of Case 5.

#### Triple-Effluent Environment

**Figure 15:**
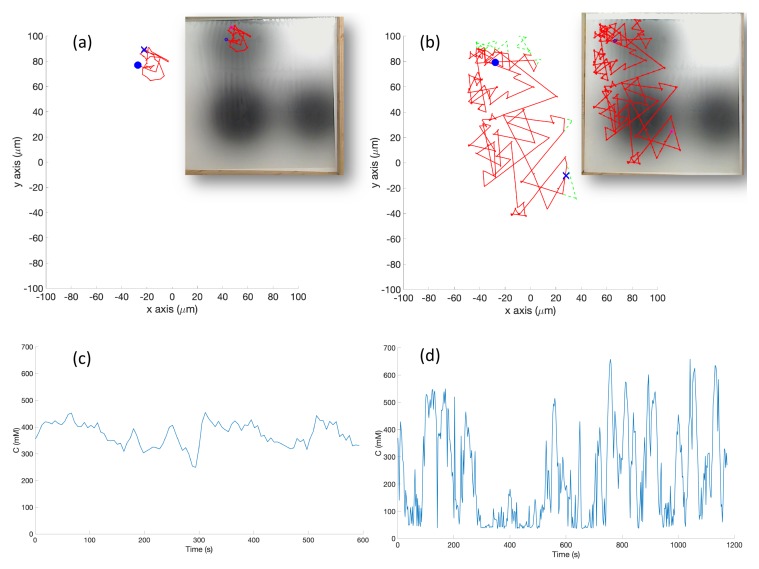
Case 6: Without obstacles, in the triple-effluent environment, typical trajectories of the e-puck robot running (**a**) the basic chemotaxis controller, and (**b**) the RapidCell chemotaxis controller. In each trajectory, the initial position is shown as a blue circle and the terminal position is shown as a blue “X”. Positions corresponding to concentrations less than 1 mM are shown in dashed green, otherwise shown in red. The recorded concentrations associated with each scheme are shown in (**c**,**d**) respectively.

**Experiments with Triple-Effluent Environment (Case 6)**: The experimental results were similar to the corresponding simulation results (Case 3). The basic controller generally traveled toward darker regions and explored the region in close proximity to the nearest peak. The RapidCell controller on the other hand explored the entire region of detectable intensity after encountering detectable chemical. Figure 15 plots one representative replicate of Case 6.

### 3.3. Simulation with Obstacles

This set of simulations adds the obstacles shown in Figure 5 to the arena.

**Figure 16:**
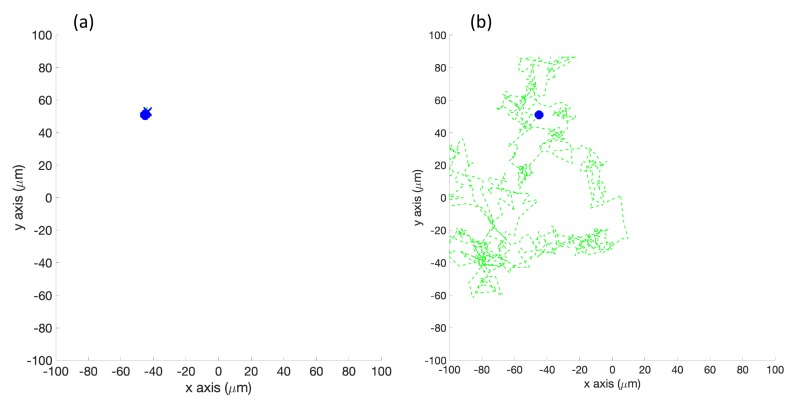
Case 7: With obstacles, in the homogeneous/no effluent environment, typical trajectories of the simulated robot running (**a**) the basic chemotaxis controller, and (**b**) the RapidCell chemotaxis controller. In each trajectory, the initial position is shown as a blue circle and the terminal position is shown as a blue “X”. Positions corresponding to concentrations less than 1 mM are shown in dashed green, otherwise shown in red.

**Simulation in Homogeneous/No Effluent Environment, with Obstacles (Case 7)**: Figure 16 shows the case where no chemical was present. Figure 16a shows that the basic controller tumbled continuously in a very small area about the initial location. Figure 16b shows that the RapidCell controller performed an unbiased random walk about the environment, encountering no chemical.

**Figure 17:**
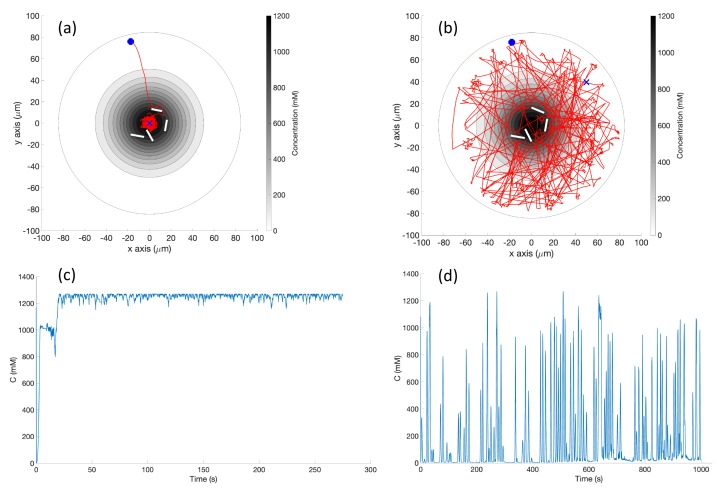
Case 8: With obstacles, in the single-effluent environment, typical trajectories of the simulated robot running (**a**) the basic chemotaxis controller, and (**b**) the RapidCell chemotaxis controller. The contour plot represents the concentration distribution shown in Figure 5 (left) where the outmost level curve represents the concentration at 1 mM. In each trajectory, the initial position is shown as a blue circle and the terminal position is shown as a blue “X”. Positions corresponding to concentrations less than 1 mM are shown in dashed green, otherwise shown in red. The recorded concentrations associated with each scheme are shown in (**c**,**d**) respectively.

**Simulation in Single-Effluent Environment, with Obstacles (Case 8)**: The basic chemotaxis controller in this situation behaved similarly to a standard hill-climbing algorithm [43]. The effluent profile is shown in Figure 3 (left). Figure 17 shows an example trajectory in this environment. The basic controller again quickly locked in on the peak and meandered in a very small area around it, with brief detours for obstacles. The larger capture region of the RapidCell controller made the detours for obstacles more obvious.

**Figure 18:**
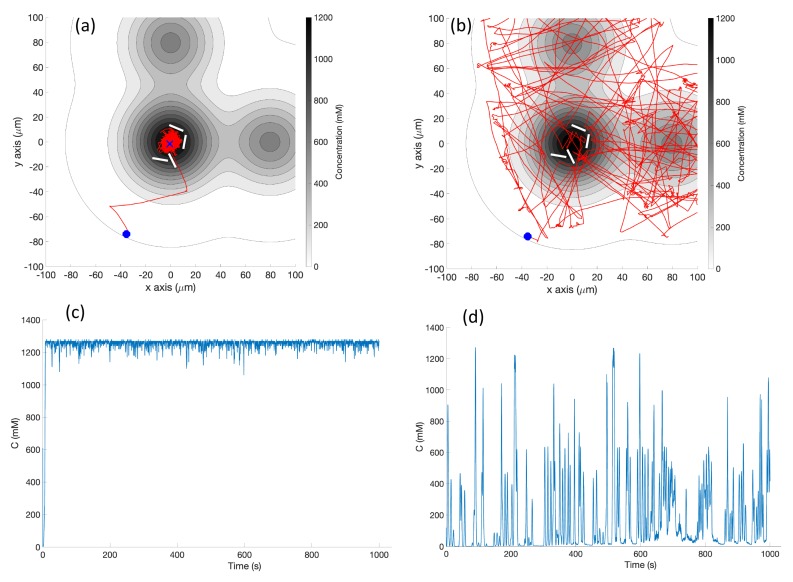
Case 9: With obstacles, in the triple-effluent environment, typical trajectories of the simulated robot running (**a**) the basic chemotaxis controller, and (**b**) the RapidCell chemotaxis controller. The contour plot represents the concentration distribution shown in Figure 5 (right) where the outmost level curve represents the concentration at 1 mM. In each trajectory, the initial position is shown as a blue circle and the terminal position is shown as a blue “X”. Positions corresponding to concentrations less than 1 mM are shown in dashed green, otherwise shown in red. The recorded concentrations associated with each scheme are shown in (**c**,**d**) respectively.

**Simulation in Triple-Effluent Environment, with Obstacles (Case 9)**: In this environment, there was a more complex profile as shown in Figure 3 (right), in addition to obstacles. Figure 18 shows an example trajectory in this environment. The basic algorithm was relatively unchanged, heading directly for the nearest peak. The RapidCell algorithm visited all three peaks while avoiding obstacles. The obstacles simply injected a new tumble-point in the trajectory, with no overall impact on the search pattern.

### 3.4. Experiments with Obstacles

As in the simulations above, four obstacles were added to the single-effluent environment poster to evaluate their impact on the physical implementations of the basic chemotaxis and RapidCell algorithms.

**Figure 19:**
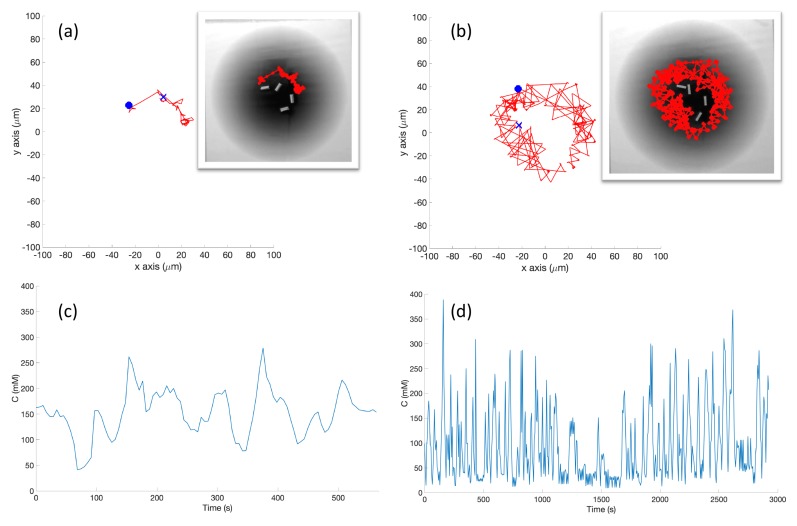
Case 10: With obstacles, in the single-effluent environment, typical trajectories of the e-puck robot running (**a**) the basic chemotaxis controller, and (**b**) the RapidCell chemotaxis controller. In each trajectory, the initial position is shown as a blue circle and the terminal position is shown as a blue “X”. Positions corresponding to concentrations less than 1 mM are shown in dashed green, otherwise shown in red. The recorded concentrations associated with each scheme are shown in (**c**,**d**) respectively.

**Experiment with Single Effluent, with Obstacles (Case 10)**: Figure 19 shows a slightly more exploratory path than seen in simulation for the basic controller (Case 8). In some replicates, this took the robot to detectable chemical. In others, it took the robot away from the chemical concentration. For the RapidCell controller, the robot’s adherence to the capture region was still noticeable, but the robot also successfully avoided the obstacles placed within the center.

**Figure 20:**
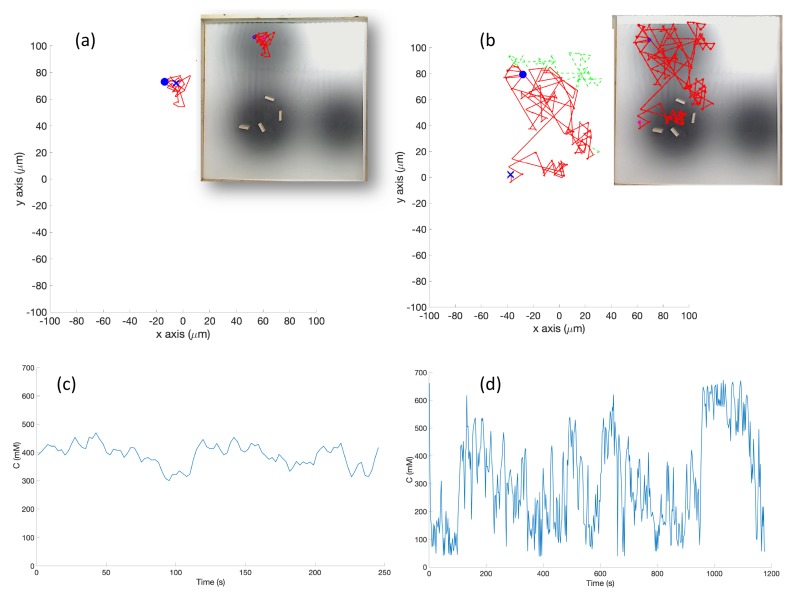
Case 11: With obstacles, in the triple-effluent environment, typical trajectories of the e-puck robot running (**a**) the basic chemotaxis controller, and (**b**) the RapidCell chemotaxis controller. In each trajectory, the initial position is shown as a blue circle and the terminal position is shown as a blue “X”. Positions corresponding to concentrations less than 1 mM are shown in dashed green, otherwise shown in red. The recorded concentrations associated with each scheme are shown in (**c**,**d**) respectively.

**Experiment with Three Effluents, with Obstacles (Case 11)**: Figure 20 shows a slightly more exploratory path than seen in simulation for the basic controller (Case 9). In some replicates, this took the robot to detectable chemical. In others, it took the robot away from the chemical concentration. For the RapidCell controller, the robot’s adherence to the capture region was still noticeable, but the robot also successfully avoided the obstacles placed within the center.

**Resulting Measurements of Success**

**Figure 21:**
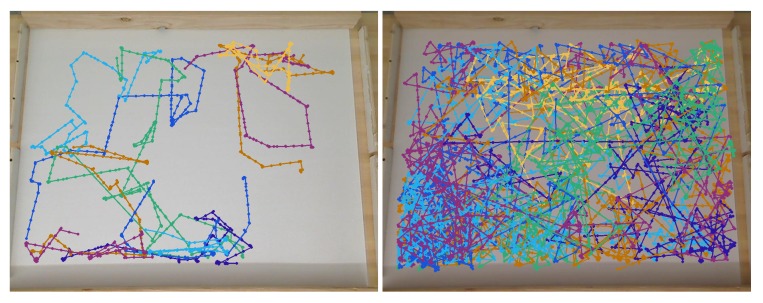
Summary of experimental runs on white poster for all 12 initial positions with (**left**) basic chemotaxis controller and (**right**) RapidCell controller. Each run is shown in a different color.

**Figure 22:**
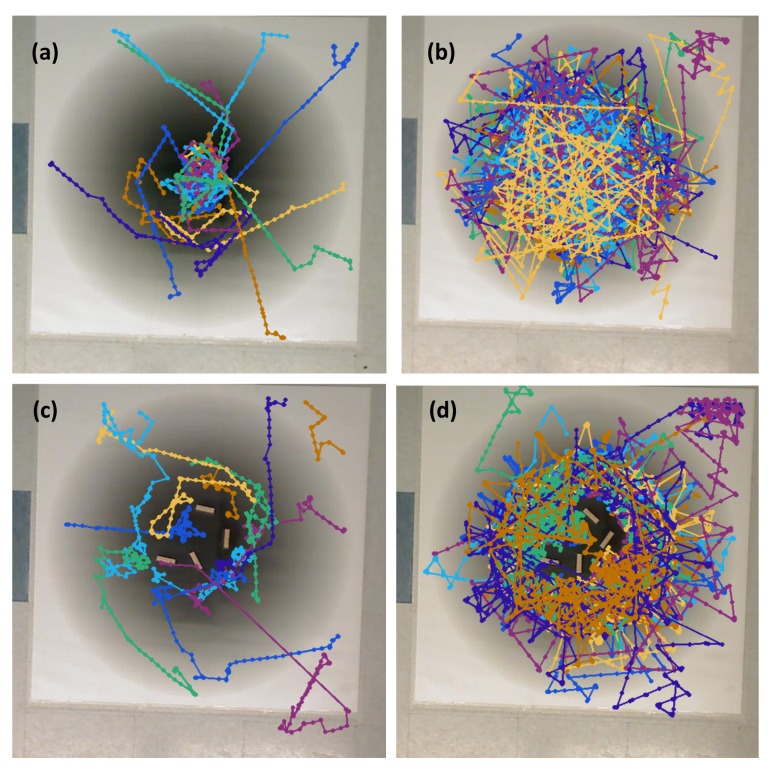
Summary of experimental runs with single effluent for all 12 initial positions that began on the area covered by the poster: (**a**) Basic controller, no obstacles; (**b**) RapidCell controller, no obstacles; (**c**) Basic controller, with obstacles; (**d**) RapidCell controller, with obstacles.

**Figure 23:**
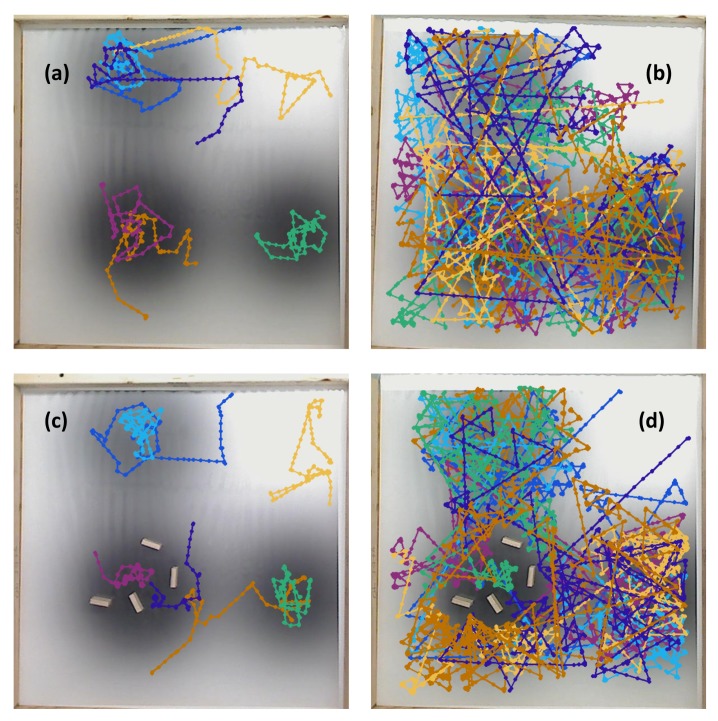
Summary of experimental runs with triple effluents for all seven initial positions that began on the area covered by the poster: (**a**) Basic controller, no obstacles; (**b**) RapidCell controller, no obstacles; (**c**) Basic controller, with obstacles; (**d**) RapidCell controller, with obstacles.

The results gave a consistent outcome—the RapidCell controller more effectively explored the regions with detectable chemical. The basic controller located the nearest peak of detectable chemical encountered, and got “stuck” there. Table 1 shows that the average percentage (AP) measure of the RapidCell controller was higher than the basic controller for all the cases, that is, the RapidCell controller traversed a much larger percentage of the region of interest than the basic controller. Figure 21, Figure 22 and Figure 23 show the trajectories associated with all valid initial positions listed in Figure 2. As a reminder, the AP measure is the average amount of the environment explored in each replicate, not a total amount over all of the replicates. It is one way to quantify the success in exploration.

When started in a region without detectable chemical, the basic controller primarily tumbles, leading to a highly localized exploration of the region thus reducing the chance of encountering the chemical plume. The RapidCell controller implements a biased random walk in this situation, increasing the success rate as shown in Table 2. The success rate (SR) reveals whether the robot was able to find the chemical concentration and remain in the capture region at the end of the simulation/experiment. Figure 21, Figure 22 and Figure 23 visually reinforce that the RapidCell algorithm was superior to the basic algorithm at more thoroughly exploring the environments in the experimental case. A similar observation was made for the simulations.

The presence of obstacles did not significantly alter these behaviors. Both controllers were able to incorporate obstacle detection and avoidance without affecting their primary behavior.

## 4. Conclusions

We presented two algorithms for mobile robots that combine chemotaxis with obstacle avoidance with the goal of exploring a region potentially containing chemical effluent while avoiding collisions with obstacles. The basic chemotaxis algorithm combines gradient ascent behavior with some random turning. The RapidCell algorithm is more biomimetic: RapidCell better simulates the behavior of real *E. coli* bacteria with a model of the intracellular signaling pathway, including a type of memory that guides the cells towards more favorable directions. We tested both algorithms in the same scenarios in simulation and experimentally with a real mobile robot platform.

We observed, both in simulation and experimentation, that the memory of the RapidCell model for chemotaxis caused a more thorough exploration of detectable chemical, without necessarily identifying the peak, while the basic chemotaxis algorithm tended to explore only around the nearest local maxima. The RapidCell controller also more fully explored homogeneous environments, increasing the chances of encountering detectable chemical. Therefore, the basic algorithm is better suited to identifying and remaining at the nearest source of chemical effluence, since it tends to get “stuck” at the first local maximum it encounters. The RapidCell algorithm is a better choice if the goal is a more thorough exploration, including in areas where there might be no detectable chemical. The presence of obstacles in the environment impeded both controllers approximately equally, not changing the qualitative behavior of either controller appreciably.

Future work includes attaching a chemical sensor that will allow the robot to determine chemical concentrations in air. In addition, a more robust and realistic simulation environment will be created using computational fluid dynamics with a gaseous attractant and the presence of moving air to more closely simulate the real-world applications of this system. We will also experiment with swarms of robots to more quickly explore the environment. Finally, various other taxis algorithms from related studies could be implemented for comparison and optimization. 

## Figures and Tables

**Table 1:** Average percentage (AP).

Case	Simulation Basic Algorithm (%)	Simulation RapidCell (%)	Experiment Basic Algorithm (%)	Experiment RapidCell (%)
No effluent without obstacles	0.04	4.13	0.24	0.86
No effluent with obstacles	0.06	3.64	-	-
Single effluent without obstacles	0.32	13.08	0.17	1.05
Single effluent with obstacles	0.31	8.31	0.14	1.10
Triple effluent without obstacles	0.56	12.02	0.13	1.04
Triple effluent with obstacles	0.56	12.81	0.14	1.11

**Table 2:** Success rate (SR).

Case	Simulation Basic Algorithm (%)	Simulation RapidCell (%)	Experiment Basic Algorithm (%)	Experiment RapidCell (%)
Single effluent without obstacles	50	100	100	100
Single effluent with obstacles	50	85	100	100
Triple effluent without obstacles	85	100	86	100
Triple effluent with obstacles	85	100	100	100

**Figure A1:**
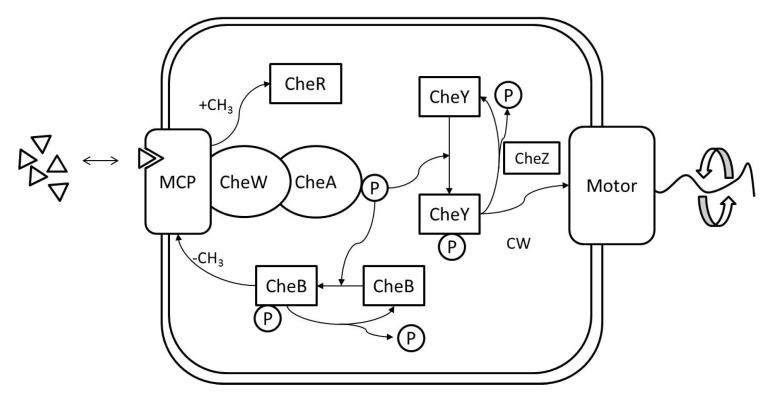
The intracellular signaling pathway of *E. coli* for chemotaxis. As the chemical molecules (represented as triangular shapes) or chemoattractants bind to the the methyl-accepting chemotaxis proteins (MCP) on the bacterial cell membrane, they activate chemical signaling among *Che* regulator proteins. *P* represents phosphoryl group, transferred among signaling components. +CH${}_{3}$ and −CH${}_{3}$ represent the methylation and demethylation, respectively, of membrane-bound chemoattractant receptor clusters. Running behavior for bacteria occurs when flagella rotate in a concerted counter-clockwise (CCW) direction; otherwise, the flagellar motor switches to clockwise (CW) rotation, resulting in tumbling behavior.

## Appendix A. RapidCell Model for Intracellular Signaling Pathway of E. coli Chemotaxis

The RapidCell model [1] was originally developed to describe the interactions of chemotaxis proteins inside *E. coli* cells, as shown in Figure A1. In this figure, chemotactic behavior is initiated through binding of chemoattractant molecules C (triangles) to the methyl-accepting chemotaxis proteins (MCP) on the bacterial cell membrane. MCP conformation changes are transmitted to the chemotaxis protein CheA through CheW. Next the phosphoryl group of CheA-P is transferred to CheY and CheB. Note that an increase of attractant inhibits the histidine kinase CheA activity. Therefore CheY is unphosphorylated which causes flagellar motors to rotate CCW (i.e., the cell runs). Meanwhile, subsequent methylation (CH${}_{3}$) at the receptor returns CheA activity to its original level: CheR adds methyl groups to the receptor (+CH3), while phosphorylated CheB removes the methyl groups (−CH3). This means that the presence of the methyl groups excites the autophosphorylation by CheA, which counteracts the effect that attractant binding has on phosphorylation. Therefore, although an *E. coli* cell tries to optimize its running mode (up the attractant gradient or down the repellent gradient), its adaptive feedback response helps to adjust its trajectory so that it can stay within a favorable environment.

In this study, we used the RapidCell model to control the behavior of chemotactic robots to detect and localize chemical sources. To describe the intracellular signaling pathway of *E. coli*, the RapidCell model involves Equations (A1)–(A6).

(A1) $$\begin{array}{c} f_{r}\left(m\right)=\epsilon_{r}\left(m\right)+ln\left(\frac{1+C/K_{r}^{off}}{1+C/K_{r}^{on}}\right)\mathrm{for}\;r=s,a \end{array}$$

(A2) $$\begin{array}{c} F=n_{a}f_{a}\left(m\right)+n_{s}f_{s}\left(m\right) \end{array}$$

(A3) $$\begin{array}{c} A_{c}=\frac{1}{1+e^{F}} \end{array}$$

(A4) $$\begin{array}{c} \left[\mathrm{CheY}-P\right]=3\frac{k_{Y}k_{s}A_{c}}{k_{Y}k_{s}A_{c}+k_{Z}{\left[\mathrm{CheZ}\right]}_{tot}+\gamma_{Y}} \end{array}$$

(A5) $$\begin{array}{c} \frac{dm}{dt}=a\left(1-A_{c}\right)\left[\mathrm{CheR}\right]-bA_{c}\left[\mathrm{CheB}\right] \end{array}$$

(A6) $$\begin{array}{c} mb=(1+\left(1/mb_{0}-1\right){\left({\left[\mathrm{CheY}-P\right]}^{H}\right)}^{-1} \end{array}$$

In these equations, *m* is receptor methylation; $f_{r}$ is the total free energy difference between “on” and “off” states of the receptor at the specific methylation level; $\epsilon_{r}\left(m\right)$ is the offset energy, linearly interpolated between the values reported in Table A1; $K_{r}^{on/off}$ is the dissociation constant for the chemoattractant in the “on” or “off” state; *F* is the total free energy difference; $f_{a}$ is the free energy difference for Tar; $f_{s}$ is the free energy difference for Tsr; Tar and Tsr are two major receptors in *E. coli*; $A_{c}$ is the probability of the cluster activity; CheY-P is the concentration of phosphorylated CheY; $k_{Y}$, $k_{Z}$, and $\gamma_{Y}$ are rate constants; $k_{s}$ is a scaling coefficient; ${\left[\mathrm{CheZ}\right]}_{tot}$ is the total CheZ concentration; *a* and *b* are methylation scaling factors; mb is the motor bias; $mb_{0}$ is the basal motor bias; and *H* is the motor Hill coefficient.

Equations (A1) and (A2) represent the total free energy difference between “on” and “off” states of the receptors and its mean field approximation, respectively. Equation (A3) calculates the activation of clusters as a function of the total free energy difference and Equation (A4) calculates the concentration of phosphorylated CheY as the CheA kinase is activated by the receptors. Equation (A5) computes methylation rate that controls the activation of receptors. Equation (A6) calculates the motor bias corresponding to the probability of the running motion of bacteria. Notice that the motor bias (mb) is generalized as inversely related to the concentration of CheY-P and its values may range from 0 to 1 with higher values corresponding to a greater likelihood of running motion of bacteria. To determine whether a cell will run or tumble, a uniform random number *x* is generated between 0 and 1; and if *x* < mb, the particle runs. Otherwise, it tumbles. The values of the parameters used in these equations are reported in Table A2. Further details regarding the description of the RapidCell model and model parameters can be found in [1].

**Table A1:** Offset energies $\epsilon_{r}\left(m\right)$ to different methylation levels *m* [1].

m	0	1	2	3	4	5	6	7	8
$\epsilon_{r}\left(m\right)$	1.0	0.5	0.0	−0.3	−0.6	−0.85	−1.1	−2.0	−3.0

**Table A2:** Descriptions and values of predetermined parameters in RapidCell model [1].

Parameter	Description	Value
$n_{a}$	Number of Tar receptors per cluster	6
$n_{s}$	Number of Tsr receptors per cluster	12
$K_{a}^{on}$	Dissociation constant in the on state of Tar receptors	0.012 μM
$K_{a}^{off}$	Dissociation constant in the off state of Tar receptors	0.0017 μM
$K_{s}^{on}$	Dissociation constant in the on state of Tsr receptors	10${}^{6}$μM
$K_{s}^{off}$	Dissociation constant in the off state of Tsr receptors	100 μM
${\left[\mathrm{CheR}\right]}_{tot}$	Total CheR concentration	0.16 μM
${\left[\mathrm{CheB}\right]}_{tot}$	Total CheB concentration	0.28 μM
${\left[\mathrm{CheZ}\right]}_{tot}$	Total CheZ concentration	*
$mb_{0}$	Basal motor bias	0.65
*H*	Motor Hill coefficient	10.3
*a*	Scaling factor for methylation	0.0625
*b*	Scaling factor for demethylation	0.0714
$k_{Z}$	Rate constant	$30/{\left[CheZ\right]}_{tot}$μM${}^{-1}$s${}^{-1}$
$k_{Y}$	Rate constant	100 μM${}^{-1}$s${}^{-1}$
$k_{s}$	Scaling coefficient	0.45μM
$\gamma_{Y}$	Rate constant	0.1 $s^{-1}$

(*) indicates that the total ${\left[\mathrm{CheZ}\right]}_{tot}$ concentration does not need to be specified explicitly since it is canceled with itself when multiplying with $k_{Z}$ in Equation (A4).

When the chemotaxis controller is started, the robot takes a large number of samples of the initial concentration of the chemical attractant and passes it through the RapidCell model. This allows the methylation, CheY-P and other parameters to adjust to the initial environment. After this initialization, the robot begins the chemotaxis control loop.
